# Supplementary material for: Affinity purification contaminants identified by cryo-EM and mass spectrometry
Source: Biosci Rep. 2026 Jun 8;46(6):BSR20260232. doi: 10.1042/BSR20260232 (PMC13259823; doi:10.1042/BSR20260232)
Supplement: Supplementary Figures S1-S2 [file BSR-2026-0232_supp.pdf]

# Affinity purification contaminants identified by cryo-EM and mass spectrometry

Emma R. Belcher[1], Steven W. Hardwick[1], Taiana Maia de Oliveira[2] and Marko Hyvönen[1]

## Supplementary figures

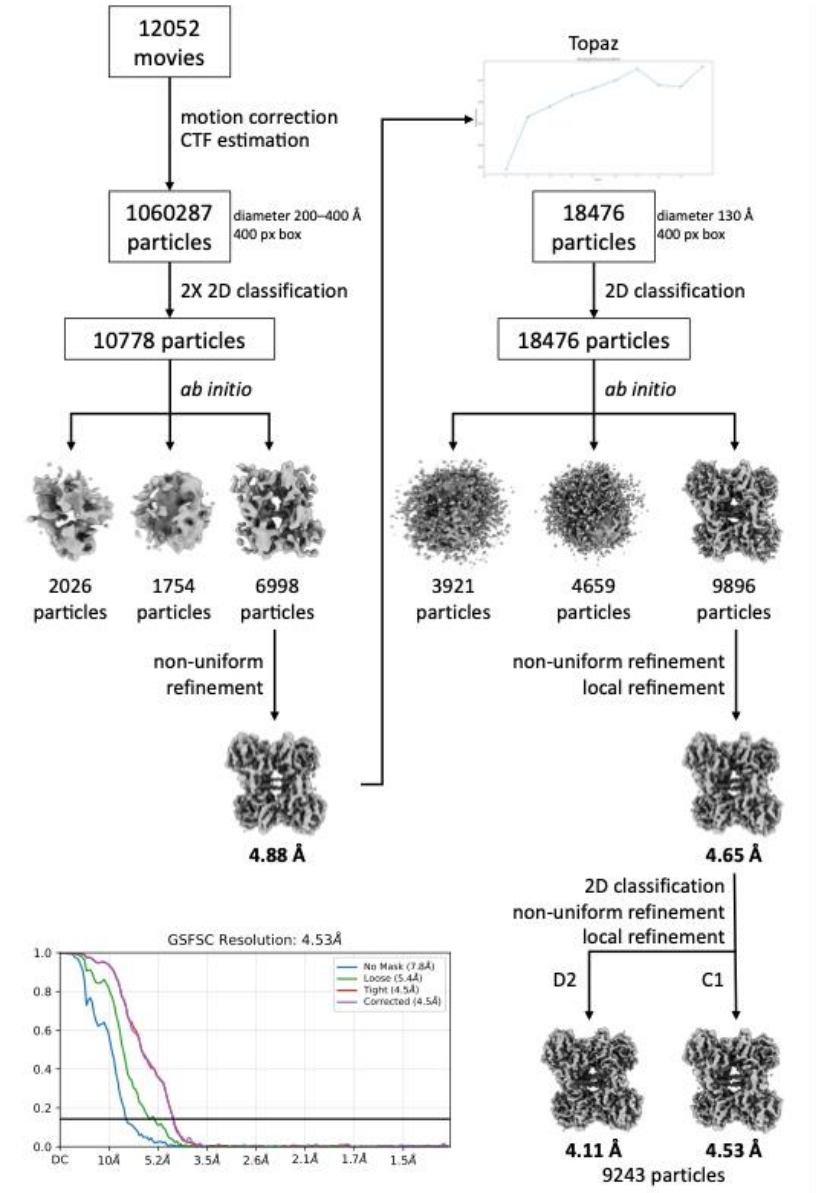


**Figure S1.** Cryo-EM data processing workflow for PRMT5:MEP50 complex. The data were processed in CryoSPARC. Volumes produced by *ab initio* reconstruction and subsequent non-uniform and local refinement are shown. Resolutions were evaluated at FSC = 0.143. After the initial volume was obtained from 6998 particles, a Topaz model was trained to search the dataset for further particles of the suspected contaminant, resulting in a final set of 9243 particles. The reported resolution of the volume was marginally improved when refined with D2 symmetry imposed. Inset: GSFSC curve of the final (C1) volume.

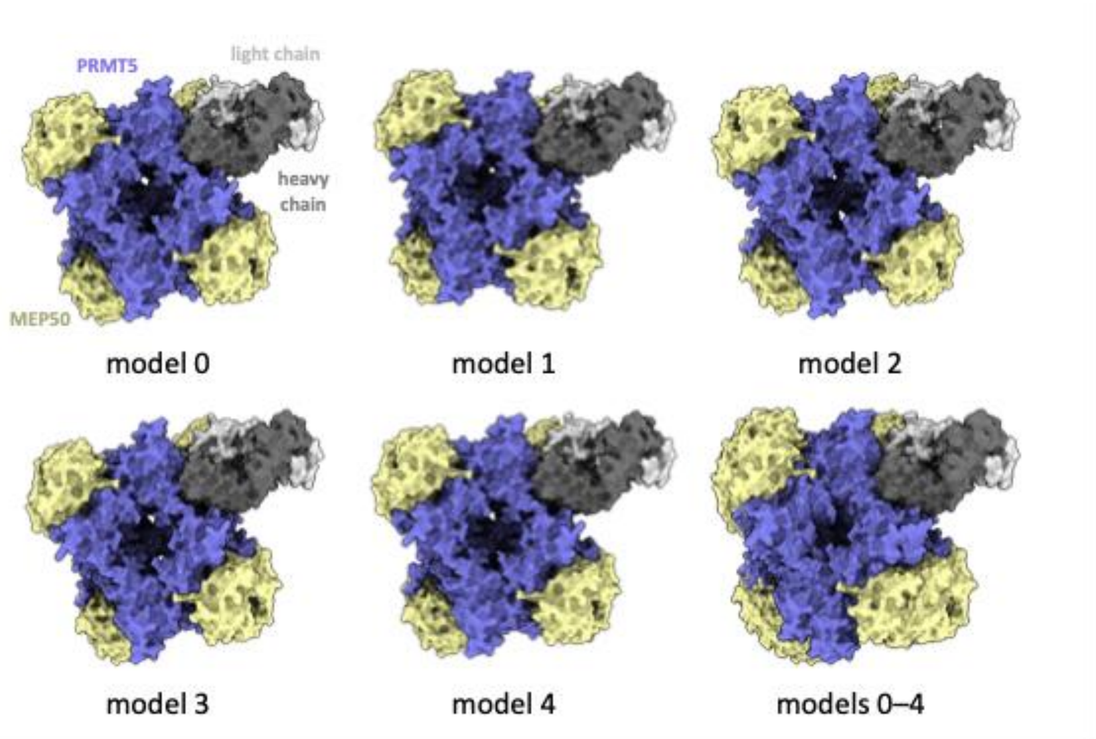


**Figure S2.** The 5 AlphaFold3 models of the anti-FLAG M2 Fab bound to protein arginine methyltransferase 5 in complex with methylosome protein 50 (PRMT5:MEP50) each show the Fab binding in a similar position to PRMT5. Four copies of PRMT5 (residues 13–637), four copies of MEP50 (residues 21–329), one copy of the anti-FLAG M2 Fab heavy chain (residues 1–217 per PDB: 8rmo), one copy of the anti-FLAG M2 Fab light chain (residues 1–219 per PDB:8rmo) and one chloride ion were included in the input. In the bottom right image, models 0–4 are shown overlaid, aligned using the heavy chain.
